# Substrate affinities of slime moulds (Eumycetozoa) and their potential as indicators of forest microhabitat conditions

**DOI:** 10.7717/peerj.21033

**Published:** 2026-05-05

**Authors:** Tomasz Pawłowicz, Tomasz Oszako

**Affiliations:** 1 Institute of Forest Sciences, Faculty of Civil Engineering and Environmental Sciences, Bialystok University of Technology, Bialystok, Podlaskie, Poland; 2 Forest Protection Department, Forest Research Institute, Sękocin Stary, Podlaskie, Poland

**Keywords:** Substrate affinities, Bioindication, Forest ecosystems, Community ecology, Bioassessment, Central and Eastern Europe

## Abstract

**Background:**

Slime moulds (Eumycetozoa) inhabit moisture-buffered microhabitats such as bark, dead wood, bryophyte mats and litter. Their assemblages are expected to track fine-scale forest structure and may therefore provide tractable signals for bioindication of forest microhabitat conditions, which represent local components of forest ecosystem condition. However, their potential as substrate-associated forest bioindicators has rarely been evaluated using harmonised data and study designs that account for sampling effort.

**Methods:**

A taxonomically standardised, georeferenced occurrence archive spanning forests in Central and Eastern Europe was used to quantify substrate affinities of slime moulds. The archive was also used to assess their suitability as ecological indicators of forest substrate (microhabitat) classes. Records were assigned to a consolidated 10-class substrate scheme representing forest substrate microhabitats where substrate descriptors were available. Analyses were restricted to these substrate (microhabitat) classes, which provided the analytical framework for all indicator assessments.

**Results:**

After sample- size- and coverage-standardisation of substrate-level record counts, record-based diversity and evenness remained high and similar across substrates. However, assemblage composition differed consistently among corticolous (bark), lignicolous (dead wood), bryophilous (bryophyte mats) and other substrates. Effort-offset multi-species modelling quantified substrate-level contrasts, whereas effort-weighted IndVal statistics identified candidate indicator taxa, with the clearest signals for lignicolous and corticolous material. Species showed and interpretable elevational modes. In contrast, pH-associated patterns were evaluated using structured screening and should be interpreted cautiously. This screening nevertheless helps to delineate preliminary pH associations and to prioritise systematic co-measurement.

**Conclusions:**

Effort-aware analyses supported consistent, substrate-associated differences in assemblage composition among forest substrate (microhabitat) classes, whereas sample-size- and coverage-standardised diversity and evenness remained broadly similar across substrates. The clearest indicator signals were recovered for lignicolous and corticolous material, with *Cribraria piriformis*, *Amaurochaete atra* and *Arcyria ferruginea* characterising lignicolous (dead wood) substrates and *Paradiacheopsis* and *Calomyxa* characterising corticolous (bark) substrates. Accordingly, substrate-resolved Eumycetozoa occurrences can inform effort-aware, regionally stratified bioassessment of forest microhabitat conditions in European forests. Given the near-absence of measured pH values in the archive, pH associations remain preliminary and systematic co-measurement of pH is a priority. Improved coverage of under-represented substrates is also required to strengthen operational Eumycetozoa-based bioindication.

## Introduction

In temperate forests, slime moulds (Eumycetozoa), as treated in contemporary classifications of eukaryotes and myxomycetes (Adl et al., 2012, 2019; Leontyev et al., 2019; Tekle et al., 2022), inhabit microhabitats such as bark, dead wood, bryophyte mats and leaf litter. Within these microhabitats, trophic stages develop within microbial biofilms and detrital matrices and sporocarps are produced episodically under suitable microclimatic conditions (Stephenson & Stempen, 2000; Stephenson, 1989). This coupling between substrate-associated trophic development and condition-dependent sporocarp expression means that substrate descriptors provide a stable, field-recordable proxy for the microhabitat conditions shaping assemblages. Substrate therefore offers a direct and operational axis for evaluating assemblage structure and potential bioindication. By grazing on bacteria and other microorganisms, these organisms contribute to nutrient turnover and help to organise detrital food webs (Keller et al., 2008; Stephenson, Schnittler & Novozhilov, 2008; Stephenson & Rojas, 2017). These life-history traits imply that substrate chemistry, porosity and microclimate shape assemblages. Consequently, corticolous, lignicolous, foliicolous, bryophilous, terricolous and other substrate contexts may sustain compositionally distinct communities (Ronikier, Ronikier & Drozdowicz, 2008; Rollins & Stephenson, 2016; Stephenson, 1989; Schnittler & Stephenson, 2000; Everhart, Keller & Ely, 2008; Schnittler, Unterseher & Tesmer, 2006; Schnittler et al., 2016; Vaz et al., 2017; Fukasawa et al., 2015).

Ecological evidence for slime moulds is commonly derived from field surveys of sporocarps. However, moist chamber cultures of bark, woody debris and litter have long been used to reveal additional taxa and to separate microhabitat limitation from episodic fruiting and detectability (de Basanta & Estrada-Torres, 2017; Schnittler, Unterseher & Tesmer, 2006; Everhart & Keller, 2008; Schnittler et al., 2016). Sporocarp production is transient and sensitive to short-term moisture pulses. Opportunistic occurrence archives can therefore over-represent readily sampled substrates and periods of favourable conditions unless sampling effort is explicitly accounted for (Stephenson & Rojas, 2017; Lado & Rojas, 2018).

The case for using slime moulds as ecological indicators of forest microhabitat conditions rests on sensitivity to substrate and microclimate. Forest management alters these properties by changing dead wood continuity, canopy cover and moisture. Such shifts are expected to be reflected in the distribution and composition of Eumycetozoa assemblages (Liu, Yan & Chen, 2015). In forests, corticolous assemblages have been shown to covary with bark pH and bark traits. In some systems, additional associations have been reported with host-tree size or vitality. In contrast, assemblages on coarse woody debris can respond to wood-decay dynamics and associated decomposer activity (Everhart, Keller & Ely, 2008; Schnittler, Unterseher & Tesmer, 2006; Schnittler et al., 2016; Vaz et al., 2017; Fukasawa et al., 2015; Takahashi & Fukasawa, 2022). Accordingly, substrate-resolved variation in taxonomic diversity and assemblage composition has potential value for bioindication of forest microhabitat conditions and for informing environmentally responsible forest management and biodiversity conservation. Such local environmental properties may contribute to broader assessments of forest ecosystem condition. Indicator development is most informative when purpose, spatial scale and validation strategy are explicit. This reduces the risk that indicator–environment relationships are assumed to be universal across contexts (McGeoch, 1998; Niemi & McDonald, 2004). Quantitative assessments across regions have nevertheless been constrained by historical data fragmentation, inconsistent nomenclature and the absence of harmonised environmental descriptors and microhabitat covariates that can be translated into consistent, auditable analytical variables across sources (notably substrate descriptors required for substrate-resolved comparisons) (Paul, Janik & Ronikier, 2023; Lado & Rojas, 2018). Progress beyond local case studies therefore depends on a workflow in which taxonomy, provenance, spatial referencing and microhabitat descriptors are standardised to enable verifiable inclusion criteria and a reproducible analysis frame. In this study, these requirements are operationalised through a Darwin Core Archive (Wieczorek et al., 2012) in which scientific names are reconciled to a harmonised taxonomy, georeferencing (where available) is retained alongside documented uncertainty and bibliographic provenance, and heterogeneous substrate descriptors are consolidated into a fixed ten-class substrateCategory scheme to enable auditable substrate-resolved analyses. Substrate-focused analyses are therefore restricted to records with non-missing species and substrateCategory, whereas pH is retained only for structured screening where measurements exist. A regional, georeferenced occurrence archive and checklist consolidating 34,588 records (1857–2025) from 16 countries in Central and Eastern Europe provides an opportunity to address historical data fragmentation under harmonised taxonomy and traceable environmental descriptors (Pawłowicz, 2025). Cross-regional evaluation of substrate-associated forest microhabitats requires consistent substrate definitions to enable like-for-like comparisons among bark, dead wood, bryophyte mats, litter and related substrates and to permit heterogeneous source descriptors to be mapped into shared substrate classes that can be used as explicit analytical strata. Because legacy occurrence data are unevenly distributed across countries and substrates, effort-aware interpretation is essential to avoid conflating non-reporting with absence and to support defensible assessment of transferable substrate-linked signals. Within such an evidence base, bioindicator potential is best understood as the reproducibility of taxon–substrate associations across regional strata when heterogeneous sampling effort is explicitly acknowledged, such that taxa can be used to indicate forest substrate (microhabitat) classes as field-recordable proxies for local microhabitat conditions relevant to forest monitoring and management. Accordingly, transferability across countries is assessed by evaluating whether substrate associations recur across country-level strata once unequal recording effort is explicitly acknowledged. In the present study, bioindicator potential therefore denotes a taxon’s capacity to provide a reproducible signal of forest substrate class for regionally stratified bioassessment of microhabitat conditions, rather than an inference about absolute abundance or stand-scale occupancy.

Although slime moulds have been widely studied, there has been no large-scale, cross-regional assessment. Most prior work has been regional, based on relatively few records, and often constrained to single forest types, individual countries, or a narrow set of microhabitats (Stephenson, 1989; Schnittler & Stephenson, 2000; Stephenson, Landolt & Moore, 1999; Schnittler, Unterseher & Tesmer, 2006; Lado & Rojas, 2018). Consequently, key uncertainties remain regarding which substrate associations are robust across countries once unequal sampling effort is considered. Further uncertainty concerns which taxa provide transferable, substrate-resolved signals suitable for forest monitoring. To address these uncertainties, the study evaluated whether assemblage composition differs systematically among major forest substrate classes when contrasts are expressed on an effort-aware scale. Uneven-effort correction reduces bias from differential recording intensity but does not, by itself, exclude compositional artefacts arising from heterogeneous survey methods, temporal coverage, or reporting practices. It further evaluated whether these contrasts persist under common sample-size and common-coverage comparisons of record-based diversity and evenness, supporting an interpretation in terms of compositional turnover. It also evaluated whether substrate signals are expressed consistently across taxonomic ranks, enabling the identification of candidate indicator taxa. Cross-rank concordance was treated as a robustness criterion, supporting indicator selection that is not driven by sparse species-level records and can be applied at the taxonomic resolution feasible for routine monitoring. Elevation was included as a complementary gradient shaping myxobiota (Ronikier, Ronikier & Drozdowicz, 2008; Rojas, Valverde & Calvo, 2016). Species-level elevational affinities were profiled as effort-adjusted descriptive summaries to support elevation-stratified interpretation of substrate signals. Measured pH values were used for structured screening where available, to delineate preliminary pH associations and prioritise systematic co-measurement.

The objective was to determine where, and at which taxonomic ranks, substrate-linked indicators show reliable performance across spatial partitions. Overall, the aim was to provide a substrate-resolved, effort-aware evidence base for the development and validation of slime mould-based indicators of forest microhabitat conditions.

## Materials and Methods

### Dataset

**Figure 1:**
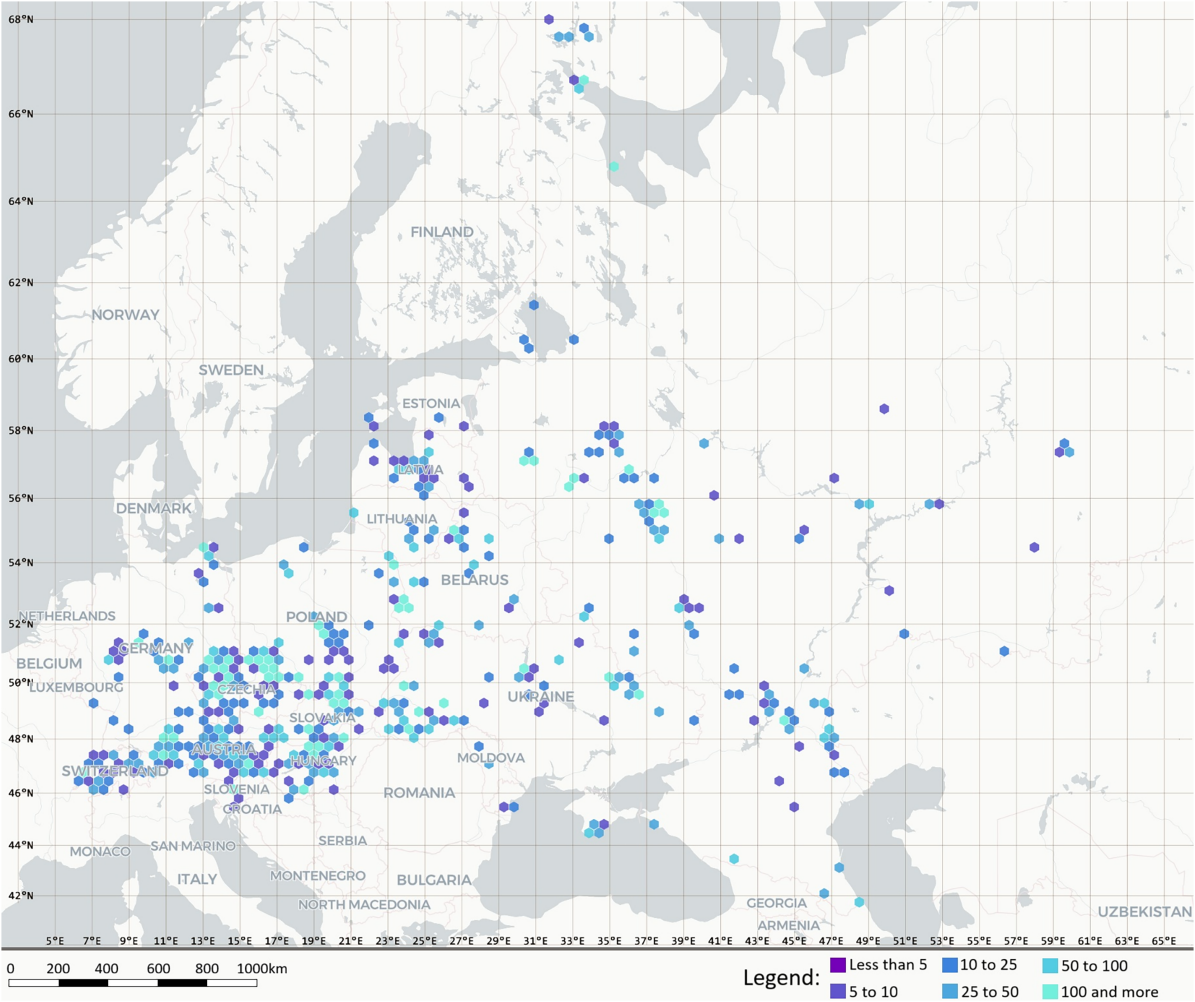
Spatial distribution of georeferenced Eumycetozoa records aggregated to 20 km hexagons (counts per cell: 0, 1–5, 6–10, 11–20, 21–50, 51–100, 101–200, >200), based on the dataset of Pawłowicz (2025). Records lacking coordinates were retained in all non-spa. Base map rendered with MapLibre GL JS and generated in kepler.gl (2025).

Analyses used a Darwin Core Archive compiling georeferenced records of slime moulds from 16 countries in Central and Eastern Europe (Pawłowicz, 2025). A taxonomically standardised regional checklist accompanied the archive. The core occurrence table contained 34,588 records (1857–2025) and included bibliographic provenance. Given the 1857–2025 observation window, data collection methods, identification resources and reporting standards are expected to have varied through time. The archive was therefore treated as a harmonised, effort-annotated presence-only resource rather than as a single standardised survey. Taxonomy was harmonised following the dataset curation framework (Pawłowicz, 2025). Accepted scientific names were reconciled primarily against Eumycetozoa.com (https://eumycetozoa.com/), with the GBIF Species backbone used as a fallback, and higher taxonomy was filled consistently. Spatial positions were provided as geographic coordinates in decimal degrees where available. Where coordinates were available, georeferencing followed the WGS84 datum and was limited to occurrence localities (geographic sampling localities) for which the locality information permitted assignment of a single point coordinate with an estimated positional uncertainty not exceeding 10 km; records not meeting this threshold were treated as lacking coordinates and were retained for non-spatial analyses, as documented for the source archive (Pawłowicz, 2025). Coordinates, where available, were used for georeferencing validation and to summarise sampling intensity on a 20 km hexagonal grid (Fig. 1); they were not used as numerical predictors in ecological models.

Core analytical variables comprised country, taxonomic identity (species and higher ranks) and substrateCategory. Topographic descriptors used in analyses included elevation bounds (minimumElevationInMeters, maximumElevationInMeters), retained as provided in the source records. Measured pH values were used only for structured screening where available. Other environmental fields (air temperature, annual precipitation and stand age) were excluded from analyses owing to sparse and non-uniform coverage and inconsistent derivation across the 1857–2025 window. Controlled vocabularies were used to define the ten substrate classes underpinning substrateCategory (Table 1). Assignment to substrateCategory was undertaken only where the source record contained an explicit substrate descriptor that could be mapped to a single class under conservative decision rules, requiring an unambiguous descriptor consistent with a single substrate class; ambiguous or mixed substrate descriptions were left unassigned (Table S1; Pawłowicz, 2025); otherwise substrateCategory remained missing (or was coded as miscellaneous (MSC) only when the source explicitly indicated an indeterminate substrate). Primary comparisons focused on the eight natural forest substrate (microhabitat) classes, with xylophilous processed wood (XYL) treated separately and saxicolous records (SAX) retained for sensitivity displays under the pre-declared rarity rule. Stand-level tree-species composition and stand structure were not considered in this substrate-focused synthesis. These drivers are addressed in a complementary study based on the same occurrence dataset (Pawłowicz, Oszako & Okorski, 2025).

**Table 1:** Consolidated substrate classification used in the study. Overarching categories, analysis codes and general definitions used to harmonise source-level descriptors for cross-regional analysis and bioindication.

Overarching substrate category	Code	General definition
Corticolous	COR	Bark of living or recently dead woody plants, encompassing the outer periderm of trunks, stems and large branches.
Lignicolous	LIG	Structural xylem tissues in any stage of decay, including logs, stumps and standing trunks of both deciduous and coniferous origin.
Ramicolous	RAM	Fine woody twigs and branches, whether still attached or fallen, generally <2 cm in diameter.
Foliicolous	FOL	Photosynthetic foliar organs (leaves, needles), either alive in the canopy or recently abscised and deposited as litter.
Bryophilous	BRY	Living moss gametophytes and moss-covered substrata.
Herbaceous	HER	Aerial parts (stems, shoots) of non-woody vascular plants, including grasses, forbs, ferns and dwarf shrubs, whether alive or senescent.
Terricolous	TER	Upper soil horizons, humus layers and surface litter composed of fragmented plant detritus on the forest floor.
Saxicolous	SAX	Exposed mineral substrata such as rocks, stones and boulders, whether bare or cryptogam-colonised.
Xylophilous (anthropogenic)	XYL	Anthropogenically processed wood (*e.g*., sawn timber, boards, wooden constructions) undergoing natural weathering and decay.
Miscellaneous	MSC	Rare, atypical or indeterminate substrata not assignable to the above categories, including unspecified decaying organic matter.

Quality control and harmonisation followed the reproducible, version-controlled Darwin Core mapping workflow, with explicit conservative criteria applied to the main uncertainty-prone steps (georeferencing retention, substrate coding and duplicate consolidation). In brief, heterogeneous sources were standardised to Darwin Core terms; scientific names were reconciled to a harmonised taxonomy; latitude/longitude fields and associated uncertainty were range-checked and retained only where locality information supported the reported positional uncertainty; and potential duplicates were screened conservatively and consolidated only when records clearly referred to the same observation event, as indicated by concordant accepted name and source provenance and, where available, consistent locality, date and substrate metadata, while retaining bibliographic provenance. Because consolidation required full concordance, some residual duplication may remain and could inflate raw record totals and the derived effort context in affected strata. Substrate descriptors were harmonised by applying conservative, deterministic decision rules anchored in Table 1: substrateCategory was assigned only when the original microhabitat descriptor permitted unambiguous mapping to a single substrate class; otherwise substrateCategory remained missing (MSC was used only when the source explicitly stated an indeterminate substrate). The original free-text microhabitat descriptor was retained *verbatim* for traceability. A derived midpoint elevation (elevationMid) was computed as the mean of minimumElevationInMeters and maximumElevationInMeters where both were available. Elevation was profiled where record counts allowed, and pH was used only for descriptive summaries and screening. For comparative analyses, records were aggregated to country × substrate × species to form a country × substrate × species record-count frame. In this design, country is treated as an administrative proxy for broad spatial structure and data provenance rather than as an ecological category. Substrate remains the microhabitat axis of inference. The total number of records within each country × substrate combination supplied the effort context for contrasts among substrates. Indicator evaluations used effort-weighted site × taxon matrices defined to the level of country × substrate and, separately, country × pH band.

Accordingly, inference concerns substrate-associated signals assessed across country-level blocks that necessarily pool heterogeneous forest contexts and sampling designs.

### Substrate classification and coding

Substrate descriptors were harmonised to the consolidated ten-class scheme (Table 1) using the controlled-vocabulary substrateCategory field (Pawłowicz, 2025). Original source descriptors were preserved in the microhabitat field for traceability, while all modelling and summaries used the harmonised classes and their two- to three-letter codes throughout (Table 1). Where the source explicitly indicated an unspecified or indeterminate substrate, the record was assigned MSC; where no substrate descriptor was available, substrateCategory remained missing, consistent with the source archive curation framework (Pawłowicz, 2025). Mapping was applied conservatively (Table S1), with assignment restricted to descriptors permitting unambiguous mapping to a single class, to reduce misclassification across languages and reporting traditions; systematic historical shifts in substrate description practice cannot be excluded and are treated as residual uncertainty in substrate-resolved comparisons. The ten-class scheme intentionally aggregates heterogeneous source terminology into broad, field-recordable microhabitat types, thereby reducing sensitivity to differences in descriptive resolution across studies, decades and languages.

### Study design, data processing, and software

Occurrence records of slime moulds were analysed to quantify substrate affinities and evaluate bioindicator potential. Indicator taxa were identified using effort-weighted IndVal (specificity and fidelity across country × substrateCategory sites) (Dufrêne & Legendre, 1997; De Cáceres & Legendre, 2009). Substrate-level contrasts were quantified using a multi-species generalised linear mixed model (GLMM) fitted to aggregated country × substrateCategory × species occurrence-record counts (y), restricted to cells with y > 0 (no absences imputed). Uncertainty was quantified by bootstrap and country-blocked validation/permutation schemes. Analyses were conducted in R using indicspecies, iNEXT, DHARMa and emmeans. Records lacking pH were retained for all substrate analyses; pH-dependent screening used only records with non-missing species, substrateCategory and pH.

### Descriptive summaries, effort, and diversity

Targeted exploratory analyses characterised records by substrateCategory and pH. The pH variable was discretised into three bands (≤5.00; 5.01–7.00; ≥7.01). For each substrate and for each substrate × pH stratum, the number of records and species was computed. These record totals quantify recording intensity within the compiled archive and were not treated as measures of organismal abundance. The number of singletons (species represented by a single record) and Good’s coverage (1—singletons/total records; Good, 1953) were then computed. For pH-resolved summaries, strata with *n* > 30 were prioritised for interpretation, while smaller strata were retained for coverage reporting. To compare the diversity structure of the occurrence archive across substrates at a common sample size and completeness, diversity, dominance and evenness were estimated after rarefaction to m = 27. Pielou’s evenness (J) was calculated as q1D/q0D (equivalently exp(H′)/S) and is bounded between 0 and 1. Rarefied diversity was obtained *via* iNEXT (estimateD, q = 0, 1, 2; 200 bootstraps) (Hsieh, Ma & Chao, 2016), and substrate-level estimates were reported with 95% bootstrap intervals. A coverage-standardised panel was included using iNEXT with base = coverage and a target coverage of 0.90 (sensitivity at 0.85) (Chao & Jost, 2012; Hsieh, Ma & Chao, 2016); Hill numbers q0D, q1D and q2D (200 bootstraps) were reported (Chao et al., 2014).

### Composition of records model frame and offset

To examine substrate effects without imputing absences, observations were aggregated to country × substrateCategory × species to yield the response y (the number of archived occurrence records of a given species in a given country × substrateCategory cell). Only observed combinations were included (y > 0); unobserved combinations were not generated as zeros and were not interpreted as absences. For each country × substrateCategory combination (treated as a site), sampling effort was computed as a leave-one-out total record count excluding the focal species (the total number of other Eumycetozoa records in the same cell). The logarithm of this leave-one-out total (with a small constant added for numerical stability) was used as an offset. Country × substrateCategory cells with near-zero exposure (<5 records) were flagged and their estimates treated as descriptive.

### Modelling substrate effects

A multi-species GLMM was fitted to aggregated country × substrateCategory × species record counts (y) and restricted to cells with y > 0 (only observed combinations; no zeros generated), using a zero-truncated negative binomial and a log link. The fixed effect was substrateCategory, with random effects specified as (1 & + substrateCategory | species) and (1 | country). A log effort offset based on the leave-one-out country × substrateCategory total (excluding the focal species) was included. Exponentiated fixed-effect contrasts (rate ratios, exp(β)) were reported relative to the reference substrate (lignicolous), with 95% confidence intervals, interpreted as relative differences in expected occurrence-record counts per unit effort conditional on a species being recorded in a country × substrateCategory site.

*Post hoc* contrasts among substrates were derived as estimated marginal means and pairwise ratios on the response scale, evaluated at unit effort (offset fixed at 0) using emmeans, with Tukey adjustment. These contrasts were used only to summarise substrate-level differences in conditional recording intensity; candidate indicator taxa were identified independently using IndVal.

Order-level deviations in substrate preferences were assessed using a separate mixed model fitted to country × substrateCategory × order record counts. SubstrateCategory was specified as a fixed effect, with contrasts defined relative to the lignicolous reference. Random effects comprised random intercepts and random substrateCategory slopes for orders (1 + substrateCategory | order), together with a random intercept for country (1 | country). Order-specific deviations are reported on the log scale with 95% intervals (parametric bootstrap or conditional variance approximation) and interpreted descriptively.

Fixed effects were interpreted as intensity ratios conditional on being recorded at a country × substrateCategory site, not as absolute abundance multipliers. Substrate categories with fewer than 100 total records were excluded from primary rankings and shown only in sensitivity analyses (SAX excluded). To reflect information imbalance across substrates, an uncertainty-aware rank display was generated from B = 2,000 parametric bootstrap replicates of the fitted GLMM, ranking estimated marginal means computed per replicate at unit effort (offset fixed at 0). The rank distribution was summarised by the median and the 50% and 95% rank intervals. As a sensitivity, information-weighted ranks were also computed using weights ws = ns/ $\Sigma{\rm s}$ ns, where ns is the total number of observations contributing to each substrate (as reported in Table 2).

**Table 2:** Uncertainty-aware substrate ranks from bootstrap GLMM estimated marginal means (offset = 0; B = 2,000): median rank (50%, 95%); information-weighted median ranks (weights by ns, n per substrate). SAX excluded (<100 records).

Substrate	Median rank	50% interval	95% interval	Info-weighted median
Xylophilous (XYL)	1	1–2	1–3	1
Terricolous (TER)	3	3–5	3–6	3
Bryophilous (BRY)	4	4–6	3–7	4
Miscellaneous (MSC)	5	5–6	4–7	5
Herbaceous (HER)	6	6–8	5–8	6
Foliicolous (FOL)	6	6–8	5–9	6
Ramicolous (RAM)	7	7–8	6–9	7
Corticolous (COR)	8	8–9	7–9	8
Lignicolous (LIG)	9	9–9	9–9	9

### Model adequacy and validation

Model adequacy of the zero-truncated negative binomial GLMM was evaluated using simulation-based residual diagnostics (DHARMa) and leave-one-country-out cross-validation (LOCO-CV) as the primary predictive check, treating country as a conservative spatial/provenance block rather than an ecological unit. Predictive performance under LOCO-CV (16 folds) was summarised by deviance and root mean squared error (RMSE). Calibration was assessed using the slope from a Poisson generalised linear model (GLM) of held-out country × substrateCategory × species record counts (y > 0) on log( ${\hat{\rm \mu}}$), with 95% confidence limits based on robust Eicker–Huber–White standard errors. Decile calibration plots display the number of observations per decile.

### Sensitivity analyses

Sensitivity to model specification and data handling was assessed by re-estimating substrate effects under four GLMM variants and by applying a pre-declared rarity rule (threshold = 100 records) for primary rankings. Effects were expressed as rate ratios (exp(β)) relative to the reference substrate, and contrasts were derived from model estimates (using emmeans when available, otherwise from fixed effects).

Robustness of pH mode estimates was examined by estimating species-level modal pH values on two grids (0.01- and 0.1-unit pH binning) using Poisson spline fits to binned counts. Modal pH estimates were interpreted cautiously where sparsity or boundary effects could influence the argmax. Concordance between the two resolutions was summarised using Pearson and Spearman correlations of per-species modal pH estimates.

### Elevational affinity profiling and indicator analyses

Species-level elevational profiles were summarised by binning occurrences into approximately 60 equal-width elevation bins and fitting per-species, effort-adjusted Poisson spline models to bin-level counts, with a log exposure equal to the total Eumycetozoa records in the same country × elevation bin. This yields effort-adjusted profiles of relative recording intensity along elevation within the compiled archive. Because exposure and bin-level counts are uneven in a heterogeneous, presence-only archive, narrow local peaks can arise from sparsity and smoothing; modal elevations are therefore treated as broad descriptive modes rather than precise ecological optima. This was calculated as follows:

(1) $${y_{cbi}} \sim {\rm Poisson}\left( {{{\rm \mu }_{cbi}}} \right),$$

${\rm log}{{\rm \mu }_{cbi}} = {f_i}\left( {{\rm ele}{{\rm v}_b}} \right) + \log \left( {{E_{cb}}} \right)$

Here, ${y_{cbi}}$ is the count for species i in country c and bin b, ${E_{cb}}$ is the total Eumycetozoa count in that bin, and ${f_i}$ is a natural cubic spline. As a cross-check, species shares ( ${y_{cbi}}$/ ${E_{cb}}$) were also computed. For each species, the modal elevation (modeElev) was defined as the argmax of the fitted relative-intensity profile on a dense grid. Uncertainty was quantified using 95% bootstrap confidence intervals (B = 2,000 parametric draws with refit). Modal elevation estimates were constrained to the observed per-species elevation domain (minimum to maximum of recorded elevations). Estimates within one grid step of a boundary were flagged as boundary cases. For indicator analyses, sites were defined as country × substrateCategory (substrate indicators) and country × pH band (pH screening), and interpreted as regionally stratified signals at this resolution.

For pH screening, sites were defined as country × pH band (≤5.00; 5.01–7.00; ≥7.01). The IndVal E statistic (Dufrêne & Legendre, 1997; De Cáceres & Legendre, 2009) was computed for taxa at species, genus and family levels with effort weights and non-parametric bootstrap intervals. Given the sparse country × pH site grid, pH IndVal is reported as screening evidence only. For substrates, presence-based, effort-weighted IndVal E was computed on a site × species matrix with sites defined as country × substrateCategory; effort weights were derived from site-level totals. Species occurring in at least three sites were retained, and significance was assessed using country-blocked permutations with Benjamini–Hochberg adjustment (Benjamini & Hochberg, 1995). Candidate indicators were defined operationally as taxa showing high IndVal E (*i.e*., jointly high specificity and fidelity) for a given substrate class across country × substrate sites, supported by bootstrap confidence intervals and country-blocked validation metrics. Consequently, very rare taxa (*e.g*., singletons or taxa confined to one or two country × substrate strata) were not evaluated as indicators because their estimated specificity and fidelity are inherently unstable at this resolution.

Non-parametric bootstrap confidence intervals were obtained by resampling sites. Country-blocked K-fold validation summarised held-out specificity (A) and fidelity (B), averaged across folds (K = 5 for substrate indicators; K = 3 for pH band indicators). To summarise taxonomic distinctiveness descriptively, presence-based indicator scores were computed for higher taxa on country × substrate sites. The indicator score was A × B (range 0–1). The substrate with the maximum score was identified per taxon and taxa were ranked. No inferential testing was undertaken for this presence-based summary.

## Results

### Data integrity and completeness

All required data fields were present in the archive (*n* = 34,588 records), although completeness varied among variables. Year (range 1857–2025) and country (sixteen countries) were complete. Spatial coordinates had 28.73% missingness for both decimalLatitude and decimalLongitude. Records lacking coordinates were retained for all non-spatial analyses and were excluded only from the sampling-intensity map (Fig. 1). Elevation fields were sparsely populated. Both minimumElevationInMeters and maximumElevationInMeters had 82.01% missingness. Species identification was missing for 0.27% of records, and 625 unique species were represented overall. Substrate descriptors were variably complete, and substrateCategory had 36.38% missingness (assigned for 22,006 records), whereas 12,582 records remained unassigned after harmonisation because an unambiguous microhabitat/substrate descriptor was unavailable. pH had 99.56% missingness, and 34,437 of 34,588 records lacked a pH value (observed range 3.35–10.00), leaving 151 measured records (0.44% of the archive).

Accordingly, the principal substrate-resolved analyses were conducted on records with non-missing species and substrateCategory, whereas pH-related analyses were restricted to the measured subset (*n* = 151) and are reported as structured screening—that is, an exploratory, pH-band indicator output used to rank candidate taxa rather than to support confirmatory inference.

### Quality control flags and essential completeness

Range checks did not detect implausible values for coordinates, pH or elevation bounds. Quality control followed the workflow described in the Materials and Methods and summarised in Table S1. Missingness flags were recorded (missing species = 94; missing substrateCategory = 12,582; missing pH = 34,437). Analyses were therefore tied explicitly to the available metadata: substrate-resolved diversity standardisation, GLMM contrasts and substrate indicator assessments were supported by the 22,006 records with assigned substrateCategory; elevational profiling used only records with non-missing elevation bounds required to compute elevationMid (≈18% of records); and pH summaries/IndVal outputs used only the measured subset (*n* = 151) and are interpreted as screening evidence. Accordingly, record totals and derived effort measures should be interpreted as record-based summaries that may include some residual duplication.

### Sampling effort, richness, and effort-standardised diversity across substrates

Record totals and observed richness without requiring pH were highest for lignicolous and corticolous substrates, followed by ramicolous and foliicolous. Xylophilous (anthropogenic) and saxicolous were comparatively sparse (see Table 3). Diversity estimated at a common sample size indicated uniformly high evenness and low dominance across substrates, with rarefied Shannon diversity spanning a narrow range. Saxicolous tended to be lowest, and miscellaneous, corticolous and xylophilous among the highest. Point estimates were tightly clustered (1 – D ≈ 0.933 to 0.955; H′ ≈ 2.83 to 3.15; J ≈ 0.96 to 0.99). Wider intervals for saxicolous reflect its small sample size.

**Table 3:** Species richness and record counts by substrate category, with the number of records with measured pH and assigned substrateCategory.

Substrate abbreviation	Records	Species	pH records n
LIG	11,411	398	53
COR	3,618	333	28
FOL	1,574	236	19
RAM	1,461	248	0
HER	1,346	203	3
MSC	967	236	28
BRY	890	186	15
TER	532	172	4
XYL	180	99	0
SAX	27	19	1

Coverage-standardised diversity at target coverage C* = 0.90 separated substrates more distinctly by richness while preserving high evenness. Lignicolous and corticolous reached the highest q0D (82 and 79). Mid-tier values occurred for ramicolous, foliicolous and bryophilous (61 to 63), whereas terricolous and herbaceous were modest (55 to 58). Hill numbers of order 1 and 2 (q1D = 12.3 to 24.1; q2D = 7.1 to 12.7) confirm an even assemblage structure across substrates. Full estimates with 95% intervals are given in Table 4.

**Table 4:** Coverage-standardised diversity at target coverage C = 0.90. Hill numbers q0D, q1D and q2D with 95% bootstrap intervals (200 replicates) from substrate-level record-count (abundance) data; entries are interpolations.

Substrate	q0D	95% CI	q1D	95% CI	q2D	95% CI
Lignicolous	82	[77–87]	24.1	[22.6–25.7]	12.7	[11.9–13.6]
Corticolous	79	[74–84]	23.3	[21.8–24.8]	12.1	[11.3–12.9]
Foliicolous	62	[58–66]	19.2	[18.0–20.4]	10.6	[9.9–11.3]
Ramicolous	63	[59–67]	19.5	[18.3–20.8]	10.8	[10.1–11.5]
Herbaceous	58	[54–62]	18.1	[17.0–19.2]	9.9	[9.3–10.6]
Bryophilous	61	[57–65]	19.0	[17.9–20.2]	10.4	[9.8–11.1]
Terricolous	55	[51–59]	17.5	[16.5–18.6]	9.6	[9.0–10.2]
Xylophilous	48	[44–52]	16.0	[15.0–17.0]	8.8	[8.2–9.5]
Saxicolous	35	[29–42]	12.3	[10.9–13.8]	7.1	[6.2–8.1]
Miscellaneous	60	[56–64]	18.7	[17.6–19.9]	10.3	[9.7–11.0]

**Note:**

CI, confidence interval.

**Figure 2:**
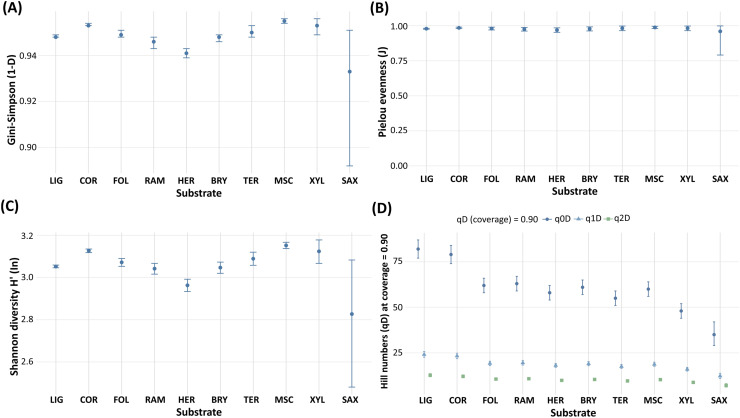
Sample-size- and coverage-standardised diversity and evenness by substrate. (A) Gini–Simpson (1–D) at a common sample size (m = 27). (B) Pielou’s evenness (J; 0–1; q1D/q0D) at m = 27. (C) Shannon diversity (H′, natural logarithm) at m = 27. (D) Coverage-standardised Hill numbers (q0D, q1D, q2D) at target coverage C* = 0.90 using iNEXT with base = coverage. Points denote estimates; whiskers show 95% bootstrap intervals (200 replicates). (D) Provides the coverage-matched comparison; (A)–(C) are sample-size-standardised summaries. Indices are derived from observed record counts per substrate (not from the GLMM offset).

Effort offsets were derived from country × substrate totals; low-effort strata were handled descriptively, and saxicolous was excluded from rankings under the pre-declared rarity rule. Sample-size- and coverage-standardised diversity and evenness by substrate are summarised in Fig. 2.

**Figure 3:**
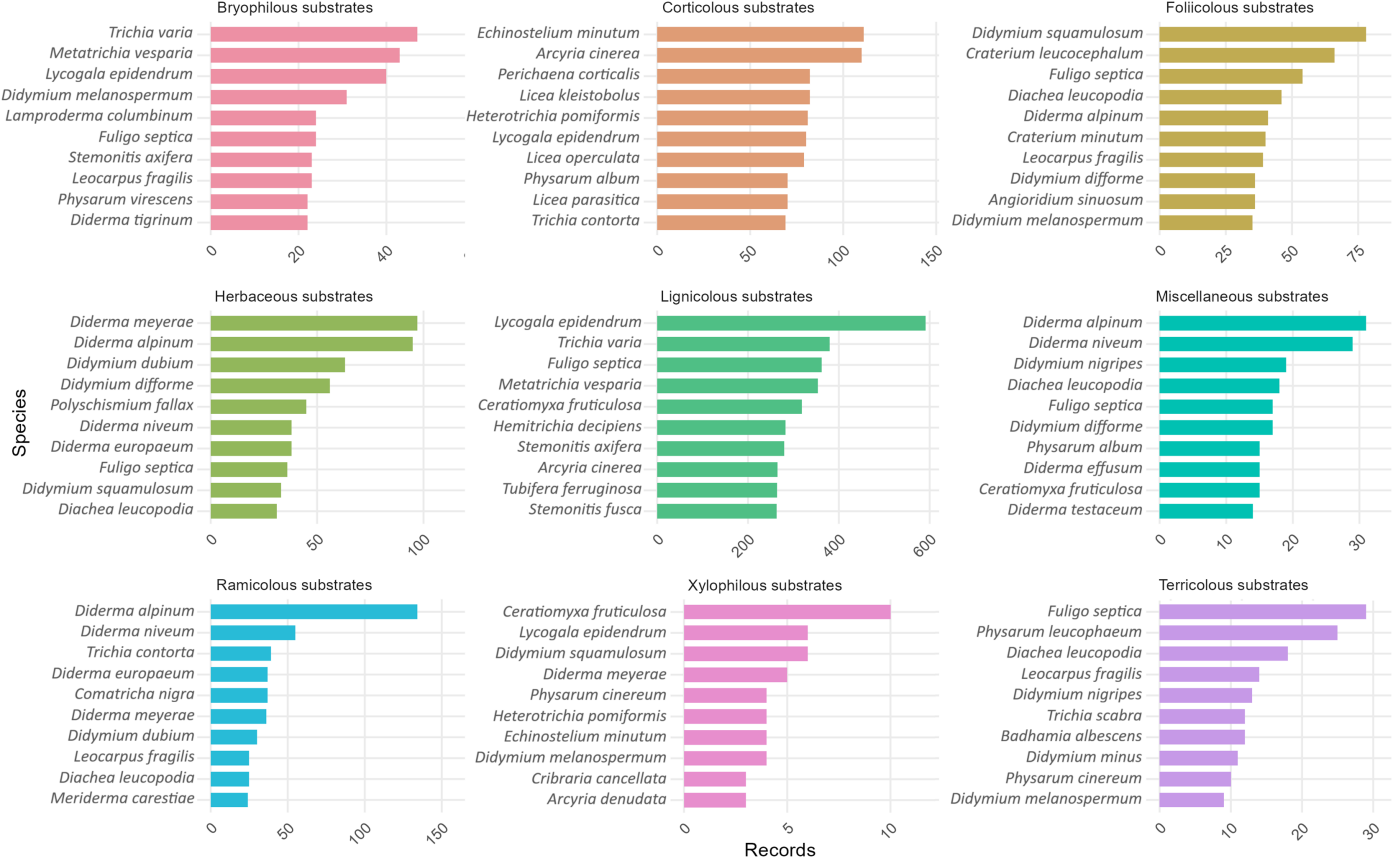
Most frequently recorded species within each substrate category. Bars show raw counts of observed occurrences; no absences were imputed. Panels display up to the ten species with the highest counts per substrate. Axis scales differ among panels, so absolute totals should not be compared across substrates.

Within each substrate category, species were ranked by observed occurrence counts to highlight the most frequently recorded taxa. In bryophilous substrates, the leading species were *Trichia varia* (Pers. ex J.F.Gmel.) Pers., 1794, *Metatrichia vesparia* (Batsch) Nann.-Bremek. ex G.W. Martin & Alexop., 1969, *Lycogala epidendrum* (L.) Fr., 1829 and *Didymium melanospermum* (Pers.) T.Macbr., 1899 (see Fig. 3). Absolute totals varied substantially between substrates, ranging from at most three records in saxicolous panels to more than five hundred in lignicolous panels (led by *L. epidendrum*). Cross-panel counts are therefore not directly comparable.

### pH distributions and coverage

Measured pH values were available for 151 records and were unevenly distributed across substrate × pH bands (Table 5). The largest stratum comprised lignicolous material at pH 5.01–7.00 (*n* = 36; Good’s coverage = 0.47), with additional mid-band observations on corticolous and foliicolous substrates (*n* = 17 each). Acidic (≤5.00) and alkaline (≥7.01) measurements were recorded across several substrates, whereas ramicolous and xylophilous records lacked pH metadata in the current archive. This sparse and unbalanced measured subset therefore provides the basis for the pH screening outputs used to flag candidate taxa and to prioritise routine co-measurement, rather than to support generalisable pH inference.

**Table 5:** Measured pH records by substrate and pH band: record totals, richness, singletons and Good’s coverage (rounded to two decimals); only strata with at least one pH record shown.

Substrate code	pH band	Records	Species	Singletons	Good’s coverage
LIG	≤5.00	6	6	6	0.00
LIG	5.01–7.00	36	25	19	0.47
LIG	≥7.01	11	10	9	0.18
COR	≤5.00	6	6	6	0.00
COR	5.01–7.00	17	15	14	0.18
COR	≥7.01	5	5	5	0.00
FOL	5.01–7.00	17	11	7	0.59
FOL	≥7.01	2	1	0	1.00
BRY	≤5.00	8	6	4	0.50
BRY	5.01–7.00	4	1	0	1.00
BRY	≥7.01	3	2	1	0.67
TER	5.01–7.00	4	3	2	0.50
HER	5.01–7.00	2	2	2	0.00
HER	≥7.01	1	1	1	0.00
MSC	≤5.00	10	9	8	0.20
MSC	5.01–7.00	9	9	9	0.00
MSC	≥7.01	9	8	7	0.22
SAX	≤5.00	1	1	1	0.00

### GLMM substrate effects, per-unit-effort means, and pairwise contrasts

**Figure 4:**
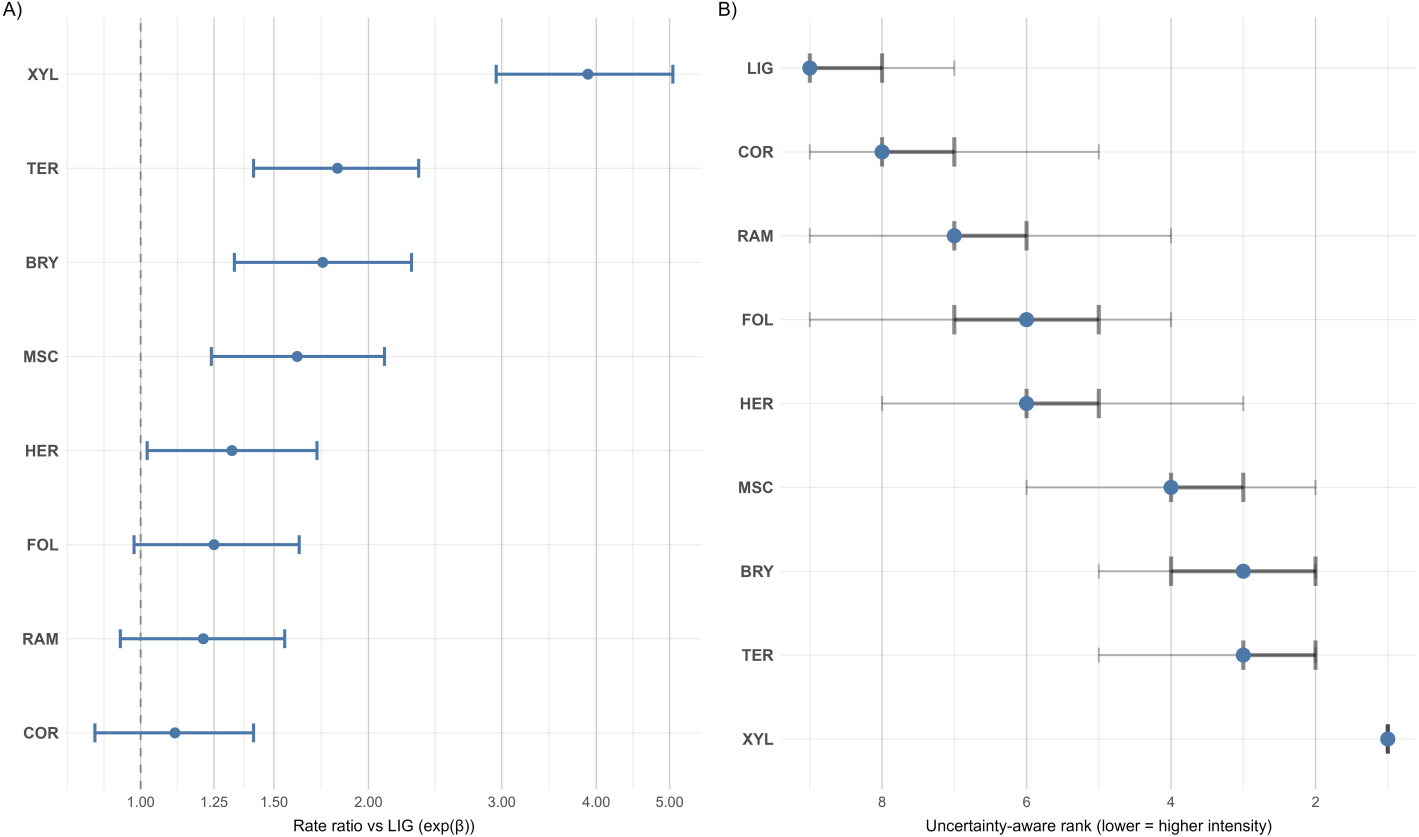
(A) Exponentiated fixed effects (rate ratios) from the zero-truncated negative binomial GLMM, reported relative to lignicolous (reference). XYL is shown for completeness. (B) Bootstrap rank uncertainty (B = 2,000): median ranks with 50% and 95% intervals; information-weighted median ranks are overlaid. SAX is omitted from the rank display by the pre-declared rarity rule.

Rate ratios from the zero-truncated negative binomial GLMM summarised conditional differences in recording intensity per unit effort among substrates (Table 6; Fig. 4). Rare categories were handled by a pre-declared rarity rule (threshold = 100 records). Consequently, the saxicolous class (SAX; total records *n* = 27) was excluded from main rankings. The xylophilous class (XYL) is anthropogenic and was reported for completeness, whereas forest-specific statements referred only to natural substrates.

**Table 6:** Substrate rate ratios *vs* lignicolous (conditional per-unit-effort) from zero-truncated negative binomial GLMM of positive country × substrate × species counts (country × substrate offset); SAX excluded (<100 records).

Substrate category	Rate ratio (exp(ß))	95% CI (lower)	95% CI (upper)
Xylophilous (XYL)	3.90	2.95	5.05
Terricolous (TER)	1.82	1.41	2.33
Bryophilous (BRY)	1.74	1.33	2.28
Miscellaneous (MSC)	1.61	1.24	2.10
Herbaceous (HER)	1.32	1.02	1.71
Foliicolous (FOL)	1.25	0.98	1.62
Ramicolous (RAM)	1.21	0.94	1.55
Corticolous (COR)	1.11	0.87	1.41
Lignicolous (LIG; ref.)	1.00	N/A	N/A

**Note:**

CI, confidence interval.

Under this specification, many non-lignicolous contrasts exceeded unity, although several 95% intervals overlapped one. Xylophilous showed a clear elevation (rate ratio 3.90 [2.95, 5.05]), terricolous and bryophilous were moderately elevated (1.82 [1.41, 2.33] and 1.74 [1.33, 2.28], respectively), and foliicolous, ramicolous and corticolous lay closer to the reference (foliicolous 1.25 [0.98, 1.62]; ramicolous 1.21 [0.94, 1.55]; corticolous 1.11 [0.87, 1.41]). SAX is retained only in sensitivity displays.

### Order-level deviations in substrate preference

**Figure 5:**
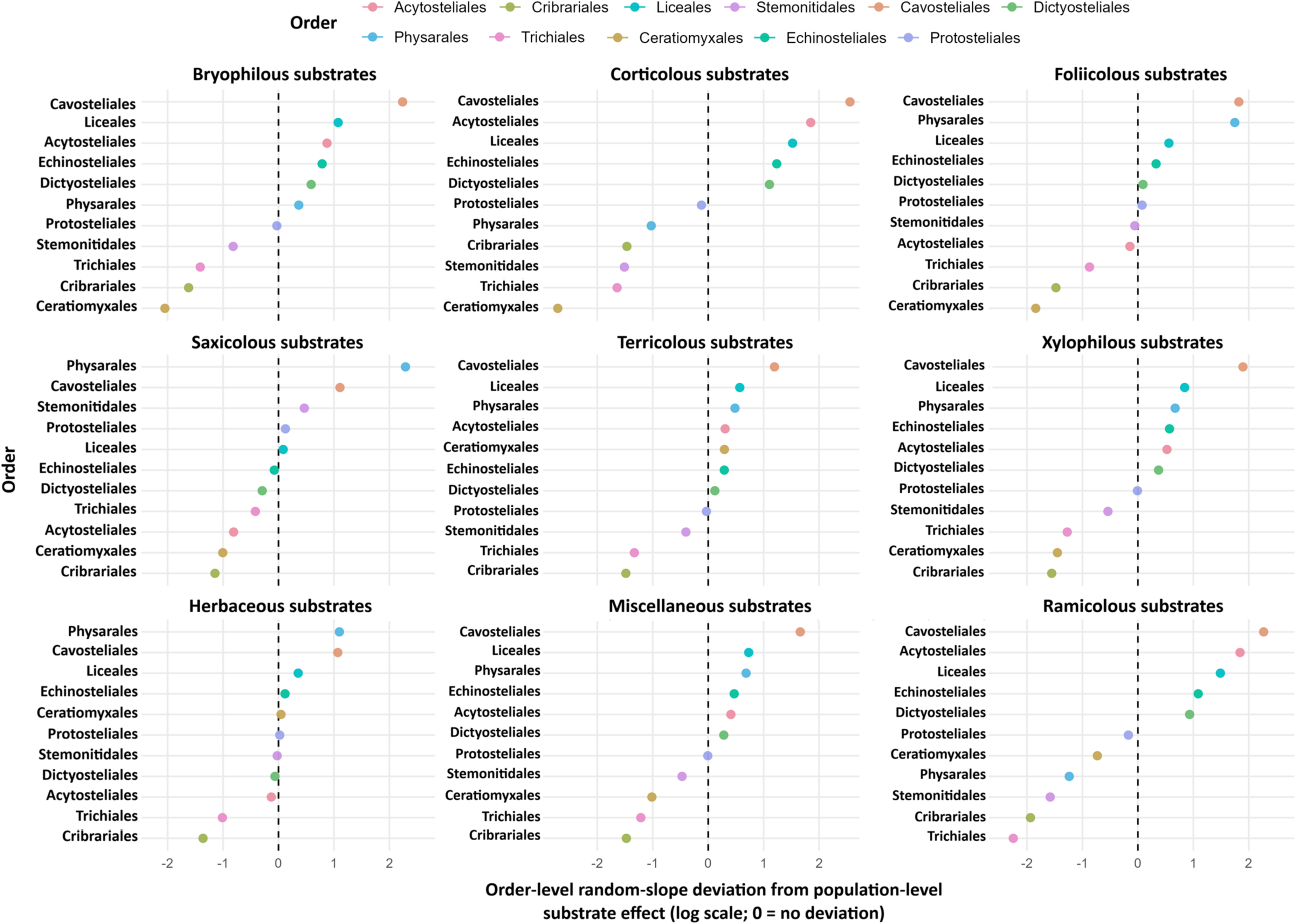
Order-level deviations in substrate preferences with partial pooling. Conditional modes (best linear unbiased predictors, BLUPs; points) and 95% uncertainty intervals (whiskers) for order-specific random slope deviations from the population-level fixed substrate effects in the order-level mixed model fitted to country × substrateCategory × order record counts. Deviations are plotted on the log scale (log ratio deviation); the vertical dashed line at 0 marks no deviation from the population-level substrate contrast (random deviation = 0). The reference substrate is lignicolous, for which no deviation parameter is estimated (panel omitted). Intervals were obtained *via* parametric bootstrap (B = 2,000) or a conditional variance approximation. Orders are sorted within each panel; directional interpretation is restricted to deviations whose 95% intervals exclude 0, and no additional multiplicity correction is applied for this descriptive display.

Order-level deviations were quantified as the conditional modes (best linear unbiased predictors, BLUPs) of the order-specific random slope terms from the order-level mixed model fitted to country × substrateCategory × order record counts (Fig. 5). These deviations are expressed on the log scale and are defined relative to the population-level fixed substrate effects; 0 denotes no deviation from the population-level substrate contrast for a given substrate (relative to the lignicolous reference). Directional interpretation was restricted to deviations whose 95% intervals excluded 0. Cavosteliales remained consistently elevated on multiple substrates (for example, corticolous: log ratio 0.61 [0.22, 1.03]), whereas Ceratiomyxales showed negative deviations on bark and twigs (for example, corticolous: –0.54 [–1.01, –0.12]). The lignicolous reference was omitted because deviations are defined relative to this reference.

### Effort-adjusted elevational affinities of representative species

Species-level elevational profiles were summarised using effort-adjusted Poisson natural cubic spline models. A log exposure equal to the total Eumycetozoa records per country × elevation bin was used, without imputing absences. The fitted curves therefore summarise relative recording intensity along elevation within the compiled archive and are conditional on the elevation distribution of available records within each country. Accordingly, narrow local peaks should be interpreted cautiously, particularly where points are sparse or exposure is uneven, and modal elevations are presented as descriptive modes rather than precise altitudinal optima.

High elevation-mode taxa include *Diderma alpinum* (Meyl.) Meyl., 1917 (1,625 m [1,510, 1,740], *n* = 316) and *Diderma meyerae* H. Singer, G. Moreno, Illana & A. Sánchez, 2003 (1,515 m [1,400, 1,640], *n* = 172). Mid-elevation modal elevations were observed for *Lycogala epidendrum* (462 m [418, 506], *n* = 133) and *Arcyria cinerea* (Bull.) Pers., 1801 (425 m [386, 468], *n* = 120). Lower elevation modes were exemplified by *Metatrichia vesparia* (380 m [342, 418], *n* = 92).

**Figure 6:**
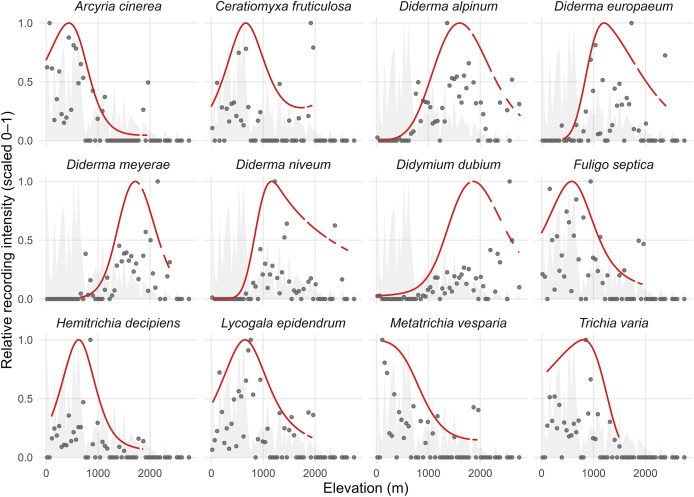
Effort-adjusted elevation profiles with uncertainty for representative species. Points show effort-normalised frequencies and lines show effort-adjusted splineGLM fits (Poisson with a log exposure equal to the total Eumycetozoa records per country × elevation bin), summarising relative recording intensity as a smooth function of elevation. Shaded ribbons denote 95% bootstrap bands (B = 2,000). Vertical ticks mark the bootstrap median modal elevation (argmax of the fitted profile) with 95% confidence-interval bars.

Additional representatives included *Cribraria piriformis* Schrad., 1797 (520 m [472, 574], *n* = 210), *Stemonitis fusca* Roth, 1787 (610 m [560, 660], *n* = 140) and *Physarum album* (Bull.) Chevall., 1826 (340 m [300, 382], *n* = 88). Representative estimates are listed in Table 7, and boundary-flagged modal elevations are marked in Fig. 6.

**Table 7:** Species elevational affinity summaries (modal elevations) with 95% bootstrap confidence intervals. Modal elevations are constrained to observed per species elevation ranges; boundary cases are flagged.

Species	*n*	Modal elevation (m)	95% CI low	95% CI high	Boundary flag
*Diderma alpinum*	316	1,625	1,510	1,740	No
*Diderma meyerae*	172	1,515	1,400	1,640	No
*Lycogala epidendrum*	133	462	418	506	No
*Arcyria cinerea*	120	425	386	468	No
*Metatrichia vesparia*	92	380	342	418	No
*Cribraria piriformis*	210	520	472	574	No
*Stemonitis fusca*	140	610	560	660	No
*Physarum album*	88	340	300	382	No

**Note:**

CI, confidence interval.

### Indicator taxa for substrates (bioindication)

Substrate-based indicator analyses were dominated by associations with lignicolous substrates. At the species rank, effort-weighted IndVal E identified taxa with high substrate specificity and fidelity. The leading species was *Cribraria piriformis* (IndVal E ≈ 0.97), followed by *Amaurochaete atra* (Alb. & Schwein.) Rostaf., 1874 and *Arcyria ferruginea* Saut., 1841 (Table 8). Permutation-based BH-FDR-adjusted values are reported for completeness and are interpreted alongside bootstrap intervals and held-out A/B metrics.

**Table 8:** Top 25 substrate indicator species (effort-weighted IndVal E): bootstrap 95% confidence intervals, BH-FDR-adjusted *p* values, and held-out specificity (A) and fidelity (B) from country-blocked validation.

Taxon	Substrate category	IndVal E score	Confidence interval lower 95 percent	Confidence interval upper 95 percent	BH FDR adjusted *p* value	Held-out specificity A	Held-out fidelity B
*Cribraria piriformis*	LIG	0.97	0.89	0.99	0.0045	1.00	0.94
*Amaurochaete atra*	LIG	0.96	0.88	0.99	0.0045	0.98	1.00
*Arcyria ferruginea*	LIG	0.95	0.86	0.98	0.0045	1.00	0.85
*Lycogala conicum*	LIG	0.94	0.82	0.99	0.0045	0.87	0.85
*Cribraria intricata*	LIG	0.94	0.84	0.99	0.0045	1.00	1.00
*Heterotrichia ferruginea*	LIG	0.94	0.85	0.98	0.0045	0.92	0.85
*Cribraria vulgaris*	LIG	0.93	0.81	0.99	0.0045	0.88	0.94
*Cribraria aurantiaca*	LIG	0.93	0.83	0.97	0.0045	0.76	0.94
*Cribraria rufa*	LIG	0.93	0.81	0.98	0.0045	0.83	0.92
*Cribraria tenella*	LIG	0.93	0.79	0.98	0.0045	1.00	0.85
*Cribraria cancellata*	LIG	0.92	0.79	0.99	0.0045	0.90	1.00
*Hemitrichia calyculata*	LIG	0.92	0.79	0.98	0.0045	0.85	0.92
*Cribraria microcarpa*	LIG	0.92	0.80	0.97	0.0045	0.73	1.00
*Cribraria argillacea*	LIG	0.91	0.75	0.97	0.0045	1.00	0.94
*Heterotrichia obvelata*	LIG	0.90	0.77	0.98	0.0045	0.93	1.00
*Metatrichia floriformis*	LIG	0.90	0.76	0.98	0.0045	0.98	0.85
*Enteridium lycoperdon*	LIG	0.90	0.76	0.97	0.0045	0.88	0.92
*Enteridium variabile*	LIG	0.90	0.74	0.97	0.0045	0.79	0.92
*Heterotrichia insignis*	LIG	0.89	0.73	0.98	0.0045	0.83	0.85
*Ceratiomyxa porioides*	LIG	0.89	0.72	0.97	0.0045	NA	NA
*Trichia subfusca*	LIG	0.88	0.69	0.97	0.0045	1.00	0.85
*Heterotrichia oerstedii*	LIG	0.88	0.66	0.97	0.0045	1.00	0.85
*Cribraria splendens*	LIG	0.88	0.64	0.95	0.0045	NA	NA
*Diderma floriforme*	LIG	0.88	0.66	0.96	0.0045	0.77	0.85
*Cribraria purpurea*	LIG	0.87	0.65	0.98	0.0045	1.00	0.94

**Note:**

N/A indicates that held-out metrics were not estimable under the blocked folds for that taxon.

At higher taxonomic ranks, presence-based indicator scores (A × B) corroborated these patterns. The highest genus-level scores were largely lignicolous, whereas *Paradiacheopsis* and *Calomyxa* represented the principal corticolous genera within the top list (Table 9). Families with the highest scores likewise separated lignicolous and corticolous alignments (Table 10). At the order level, Ceratiomyxales showed the highest score on lignicolous substrates (score = 0.33; A = 0.38; B = 0.88), followed by Liceales (lignicolous) and Echinosteliales (corticolous) (Table 11).

**Table 9:** Top 10 genera by presence-based indicator score (A × B) across country × substrate sites; substrate shown is the class with the maximum score for each genus.

Substrate category	Taxon	Presence in focal substrate count	Total presence across substrates count	Number of sites	Specificity A	Fidelity B	Indicator score A times B
LIG	*Reticularia*	15	33	16	0.45	0.94	0.43
COR	*Paradiacheopsis*	12	23	15	0.52	0.80	0.42
LIG	*Tubifera*	14	34	16	0.41	0.88	0.36
LIG	*Amaurochaete*	12	26	16	0.46	0.75	0.35
LIG	*Dictydiaethalium*	11	22	16	0.50	0.69	0.34
LIG	*Enerthenema*	12	27	16	0.44	0.75	0.33
LIG	*Ceratiomyxa*	14	37	16	0.38	0.88	0.33
COR	*Calomyxa*	9	17	15	0.53	0.60	0.32
LIG	*Enteridium*	12	29	16	0.41	0.75	0.31
LIG	*Metatrichia*	14	40	16	0.35	0.88	0.31

**Table 10:** Top 10 families by presence-based indicator score (A × B) across country × substrate sites; substrate shown is the class with the maximum score for each family.

Substrate category	Taxon	Presence in focal substrate count	Total presence across substrates count	Number of sites	Specificity A	Fidelity B	Indicator score A times B
LIG	Dictydiaethaliaceae	11	22	16	0.50	0.69	0.34
LIG	Ceratiomyxaceae	14	37	16	0.38	0.88	0.33
COR	Hemitrichiaceae	15	52	15	0.29	1.00	0.29
COR	Dianemataceae	9	20	15	0.45	0.60	0.27
COR	Echinosteliaceae	11	30	15	0.37	0.73	0.27
LIG	Tubiferaceae	16	67	16	0.24	1.00	0.24
LIG	Cribrariaceae	14	57	16	0.25	0.88	0.21
LIG	Reticulariaceae	4	5	16	0.80	0.25	0.20
COR	Liceaceae	12	51	15	0.24	0.80	0.19
LIG	Arcyriaceae	15	84	16	0.18	0.94	0.17

**Table 11:** Top 10 orders by presence-based indicator score (A × B) across country × substrate sites; substrate shown is the class with the maximum score for each order.

Substrate category	Taxon	Presence in focal substrate count	Total presence across substrates count	Number of sites	Specificity A	Fidelity B	Indicator score A times B
LIG	Ceratiomyxales	14	37	16	0.38	0.88	0.33
LIG	Liceales	4	5	16	0.80	0.25	0.20
COR	Echinosteliales	11	42	15	0.26	0.73	0.19
LIG	Cribrariales	16	85	16	0.19	1.00	0.19
COR	Trichiales	15	99	15	0.15	1.00	0.15
COR	Stemonitidales	15	105	15	0.14	1.00	0.14
LIG	Physarales	16	124	16	0.13	1.00	0.13
FOL	Protosteliales	1	1	14	1.00	0.07	0.07
TER	Acytosteliales	1	2	12	0.50	0.08	0.04
TER	Dictyosteliales	1	2	12	0.50	0.08	0.04

Overall, lignicolous genera dominated the top genus list (8/10). The estimand is occupancy and specificity across country × substrate sites (IndVal E), which is complementary to the GLMM conditional intensity.

### Indicator taxa for pH bands (bioindication)

Given the current availability of measured pH values (99.56% missing) and the limited representation of the country × pH site grid, pH associations are presented as structured screening evidence. IndVal E screening was used to summarise candidate associations with the three pH bands and to support prioritisation of systematic co-measurement. Summary estimates, BH-adjusted values and held-out specificity (A) and fidelity (B) from country-blocked validation are reported in Table 12.

**Table 12:** Indicator taxa for pH bands (screening): IndVal E with bootstrap 95% confidence intervals and held-out metrics (A, B) from three-fold country-blocked cross-validation.

Taxonomic rank	Taxon	pH band	IndVal E	BH FDR	CI lower 95 percent	CI upper 95 percent	Held-out specificity A	Held-out fidelity B
Species	*Paradiacheopsis solitaria*	5.01–7.00	0.73	0.57	N/A	0.97	0.88	0.91
Species	*Comatricha nigra*	5.01–7.00	0.65	0.57	0.57	0.96	0.26	0.41
Species	*Perichaena luteola*	≥7.01	0.64	0.57	0.32	0.95	0.00	0.00
Species	*Trichia contorta*	5.01–7.00	0.63	0.57	N/A	0.97	0.26	0.41
Species	*Comatricha pulchella*	5.01–7.00	0.60	0.57	0.44	0.96	0.26	0.41
Genus	*Stemonitis*	5.01–7.00	0.92	0.56	0.69	1.00	0.96	0.90
Genus	*Didymium*	5.01–7.00	0.80	0.56	0.63	0.99	0.84	0.90
Genus	*Licea*	5.01–7.00	0.74	0.56	0.56	0.94	0.80	0.90
Genus	*Comatricha*	5.01–7.00	0.73	0.56	0.56	0.97	0.64	0.90
Genus	*Paradiacheopsis*	5.01–7.00	0.73	0.56	N/A	0.97	0.88	0.91
Genus	*Diderma*	≤5.00	0.66	0.56	0.50	1.00	0.19	0.50
Genus	*Lamproderma*	≤5.00	0.65	0.56	0.50	1.00	0.19	0.50
Genus	*Trichia*	5.01–7.00	0.63	0.56	N/A	0.97	0.26	0.41
Genus	*Lycogala*	≤5.00	0.58	0.56	N/A	0.93	0.00	0.00
Genus	*Physarum*	≥7.01	0.56	0.56	0.43	0.91	0.00	0.00
Genus	*Arcyria*	≥7.01	0.54	0.56	0.50	1.00	0.00	0.00
Genus	*Perichaena*	5.01–7.00	0.49	0.56	0.48	0.86	0.00	0.00
Family	Liceaceae	5.01–7.00	0.74	0.58	0.56	0.94	0.80	0.90
Family	Trichiaceae	5.01–7.00	0.72	0.58	0.56	0.92	0.72	0.90
Family	Didymiaceae	5.01–7.00	0.70	0.58	0.56	0.90	0.68	0.90
Family	Stemonitidaceae	5.01–7.00	0.67	0.58	0.59	0.87	0.56	0.90
Family	Tubiferaceae	≤5.00	0.58	0.58	N/A	0.93	0.00	0.00
Family	Physaraceae	5.01–7.00	0.55	0.58	0.48	0.91	0.03	0.25

**Note:**

N/A indicates that a confidence bound or held-out metric was not estimable for that taxon given the available country × pH site grid.

At the genus rank, *Stemonitis* and *Didymium* show the strongest mesophile-band signal in the screening output, with additional support for *Licea*, *Comatricha* and *Paradiacheopsis*. At the species rank, *Paradiacheopsis solitaria* (Nann.-Bremek.) Nann.-Bremek., 1975 shows the most stable mesophile-band signal (held-out A = 0.88; B = 0.91). Additional mesophile-band candidates include *Comatricha nigra* (Pers. ex J.F.Gmel.) J.Schröt., 1885, *Trichia contorta* (Ditmar) Rostaf., 1875, and *Comatricha pulchella* (C. Bab.) Rostaf., 1876 (Table 12). At the family level, mid-band alignment is recorded for Liceaceae, Trichiaceae, Didymiaceae and Stemonitidaceae, while Tubiferaceae and genus Arcyria show screening support for lower and higher pH, respectively, within the measured subset.

### Model diagnostics, predictive calibration, and sensitivity

**Figure 7:**
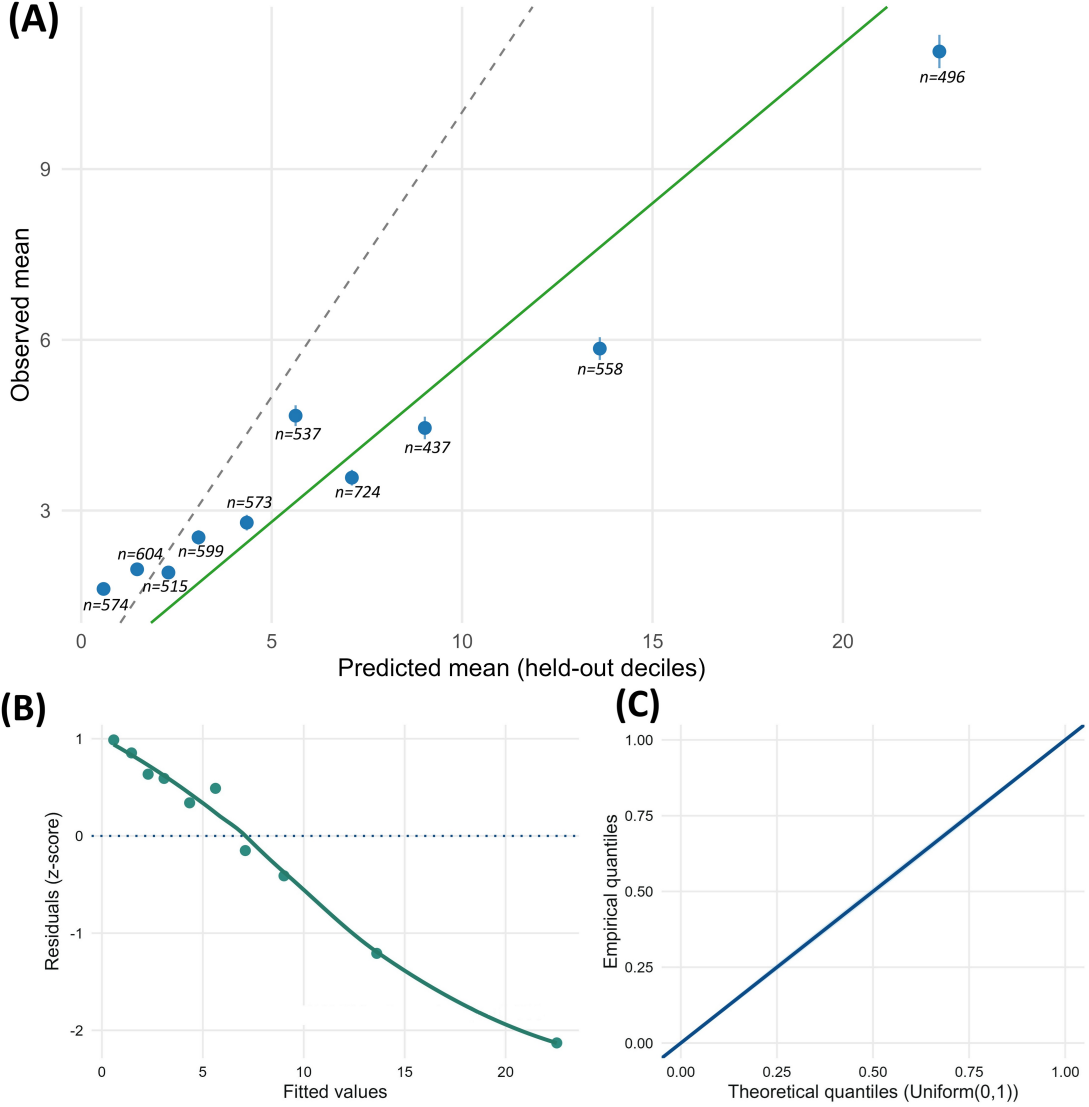
Model diagnostics and predictive calibration (LOCO-CV, 16 folds). (A) Decile calibration of held-out predictions (dashed diagonal: perfect calibration); inset: calibration slope ${\rm \beta}^{1}$ = 0.56 (95% CI [0.52–0.60]). (B) Residuals versus fitted values (z-scaled residuals, with a smooth trend). (C) Uniform(0, 1) Q–Q plot of DHARMa residuals; the diagonal indicates the expected 1:1 relationship between theoretical and empirical quantiles.

Simulation-based diagnostics (panel B in Fig. 7) show z-scaled residuals plotted against fitted values and a Uniform(0, 1) Q–Q plot. Both indicate mild curvature and a right-tail deviation without major violations. DHARMa dispersion tests supported the assumed dispersion structure (*p* = 0.333). Because zeros were structurally absent by design, zero-inflation diagnostics were not applicable in this analysis frame. Fold-wise LOCO-CV summaries are given in Fig. 7 and Table 13.

**Table 13:** LOCO-CV predictive diagnostics (primary). Mean across 16 folds (each holding out one country).

Metric	Mean	95% CI low	95% CI high
Calibration slope ${\rm \beta}^{1}$	0.56	0.52	0.60
RMSE	8.12	7.84	8.41
Poisson deviance	11,850	11,320	12,390

**Note:**

CI, confidence interval; RMSE, root mean squared error.

Under LOCO-CV (16 folds), the mean calibration slope was 0.56 (95% confidence interval (CI) [0.52–0.60]), the mean RMSE was 8.12, and the mean Poisson deviance was 11,850. Decile calibration points, labelled with *n* per decile, lay mostly below the 1:1 line at higher predictions, consistent with the estimated slope.

Country-blocked calibration (Fig. 7) shows slopes below unity, with observed decile means predominantly below the 1:1 line. Fold-wise diagnostics for the no-slopes variant (V2) under the K = 3 country-blocked scheme are reported in Table S2, and model wise means for both specifications under the same scheme are summarised in Table S3.

**Figure 8:**
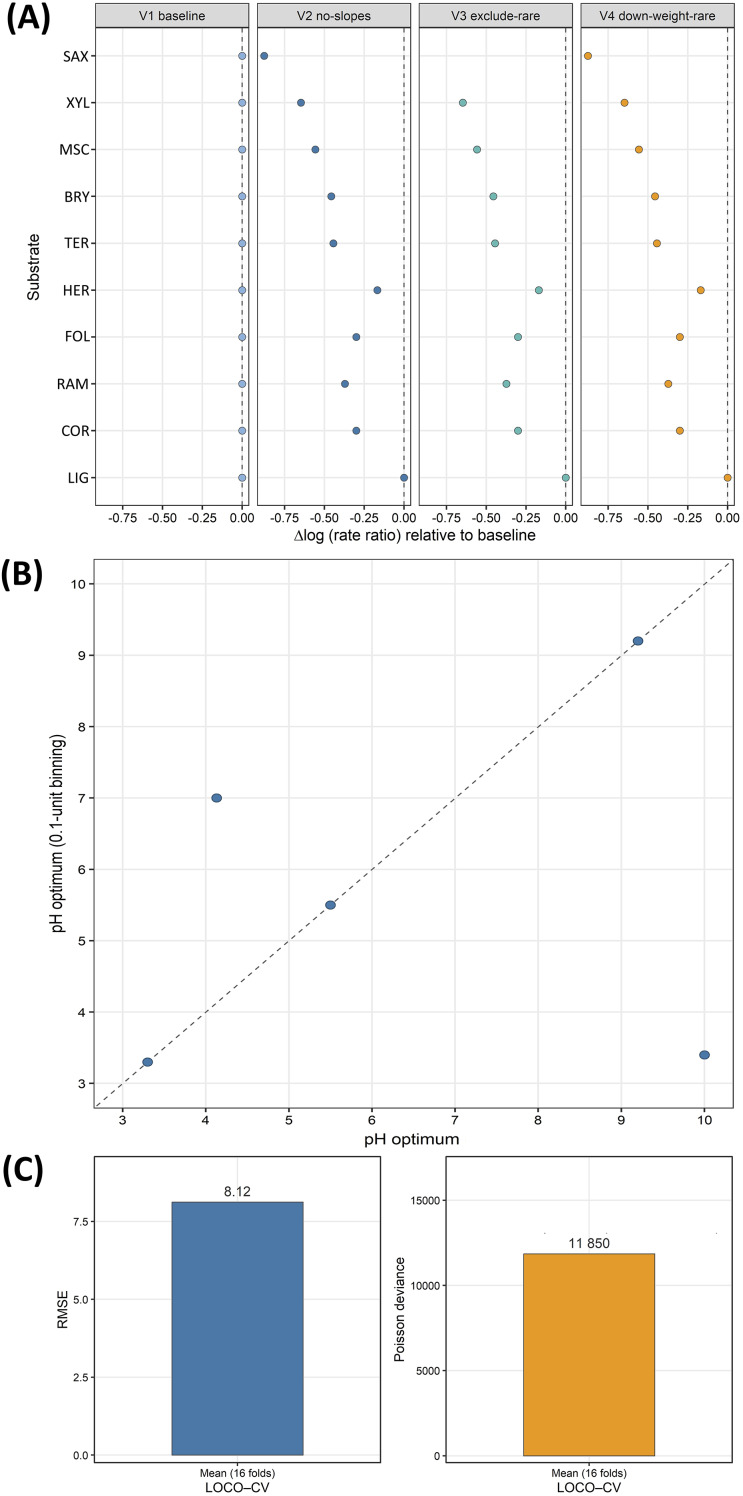
Sensitivity and robustness overview. (A) Δlog(rate ratio) for each substrate relative to the baseline GLMM across model variants (V1: baseline with species level random slopes; V2: no slopes; V3: exclude rare, threshold = 100 records; V4: down weight rare). Points indicate the median change; horizontal whiskers denote 95% confidence intervals. The vertical dashed line at 0 marks no change *vs* baseline. (B) Per species pH optima under two resolutions (two decimal rounding *vs* 0.1 unit binning) for six species; four cases are identical across resolutions. The diagonal dashed line shows the 1:1 relation (no shift between resolutions). (C) Predictive diagnostics under leave one country out cross validation (16 folds): bars display the mean RMSE (8.12) and the mean Poisson deviance (11,850); the overlaid text reports the calibration slope ${\rm \beta}^{1}$ = 0.56 (95% CI [0.52–0.60]).

Sensitivity analyses indicated consistent qualitative shifts across alternative GLMM specifications. Relative to the baseline model with species level random slopes and country intercepts, Δlog(rate ratio) values were negative for all non reference substrates. These shifts were closely aligned across the no slopes, exclude rare and down weight rare variants; the multiplicative change in rate was approximately 0.52 for xylophilous, 0.63 for bryophilous and 0.64 for terricolous. Saxicolous was identified as rare (27 records) and removed under the exclude rare variant (threshold = 100 records). Where estimable (V2 and V4), its multiplicative change was ≈ 0.42. These sensitivity patterns are summarised in Fig. 8.

Cross-resolution concordance of per-species pH mode estimates (Fig. 8B) was mixed: four of six species had identical modal pH estimates, whereas *Lycogala epidendrum* shifted by +2.87 pH units and *Lamproderma columbinum* (Pers.) Rostaf., 1873 shifted by −6.60 pH units between resolutions; correlations were modest (Pearson r ≈ 0.37; Spearman ρ ≈ 0.35).

## Discussion

### Assemblage structure and sampling context

If pH records were set aside because so many records lacked a pH reading, lignicolous and corticolous substrates dominated in raw record number and observed richness. Several other substrates were comparatively less represented (see Table 3). These totals describe the quantity of archived occurrence records (and hence the information balance among substrate groups) rather than ecological abundance. As records span 1857–2025, sampling approaches and metadata quality are expected to vary. The pooled contrasts reported here should therefore be interpreted as long-term, effort-adjusted substrate associations rather than time-resolved trends. This pattern is consistent with evidence that dead wood, bark and canopy twigs represent primary slime moulds microhabitats in forest ecosystems (Stephenson, 1989; Schnittler & Stephenson, 2000; Schnittler, Unterseher & Tesmer, 2006; Everhart & Keller, 2008; Fukasawa et al., 2015). After standardising substrate-level record counts by sample size and coverage, record-based diversity and evenness remained uniformly high across substrates (see Fig. 2; 1 − D ≈ 0.933–0.955; H′ ≈ 2.83–3.15; J (0–1) ≈ 0.96–0.99). Point estimates separated only modestly, with a consistently lower bound for saxicolous. These patterns indicate that, under comparable standardised sampling, assemblages were broadly even and diverse. Record distribution nevertheless remained uneven among substrates, shaping the leverage for inference.

Sporocarps represent an operationally tractable unit for monitoring. However, they capture fruiting activity rather than direct measures of plasmodial abundance. Consequently, effort-aware contrasts and explicit validation are required if recorded occurrences are to be interpreted as indicators of substrate context or microclimatic change (McGeoch, 1998; Niemi & McDonald, 2004). Standardised contemporary sampling would further support evaluation of temporal stability.

### Effort standardised contrasts and their implications

With a leave-one-out effort offset and a model for positive country × substrateCategory × species record counts (y > 0), substrate contrasts were expressed on a conditional, per-unit-effort scale. Non-lignicolous categories generally exceeded the lignicolous reference; among natural forest substrates, terricolous and bryophilous were moderately elevated, whereas corticolous lay closest to parity (Table 6; Fig. 4). Such contrasts are plausibly linked to systematic differences among substrates in moisture buffering, surface chemistry and decay-related resource structure. These factors have been shown to influence corticolous and lignicolous assemblages in independent forest studies (Everhart, Keller & Ely, 2008; Fukasawa et al., 2015; Schnittler et al., 2016; Vaz et al., 2017).

Blocked validation and uncertainty-aware ranking supported interval- and rank-based interpretation, and saxicolous was retained descriptively under the pre-declared rarity rule.

### Higher taxon composition and substrate signal

Order-level heterogeneity in substrate association was summarised using order-specific random slope deviations (BLUPs/conditional modes) from the population-level fixed substrate effects in the order-level mixed model (Fig. 5). These deviations are expressed on the log scale, with 0 denoting no deviation from the population-level substrate contrast relative to the lignicolous reference. Cavosteliales displayed consistently positive deviations on multiple substrates, whereas negative deviations were frequent for Ceratiomyxales, Trichiales and Cribrariales on selected substrates. Directional interpretation was restricted to deviations whose 95% intervals excluded 0, whereas intervals spanning 0 were treated as compatible with the population-level substrate effect given the available evidence.

### Substrate-based bioindicators: candidates and regionalisation

Presence-weighted scores identified characteristic lignicolous and corticolous assemblages across orders, families and genera, with high scores arising from substantial specificity and within-substrate occupancy (Tables 9–11). At the genus rank, lignicolous indicators predominated, while *Paradiacheopsis* and *Calomyxa* aligned with corticolous material within the top list (Table 9). When inference was introduced *via* effort-weighted IndVal E with country-blocked resampling, several taxa showed strong substrate alignment (Table 8). These included *Cribraria piriformis* (IndVal E ≈ 0.97), *Amaurochaete atra* and *Arcyria ferruginea*. The divergence between strong within-fold performance and geographically variable signal pointed to spatial heterogeneity in substrate associations. For application, indicator sets should therefore be regionally stratified and validated under blocked resampling, with performance reconfirmed under the same scheme.

This emphasis on explicit validation is aligned with established indicator-species methodology and broader guidance on ecological indicators, in which scale, purpose and uncertainty must be specified to avoid over-interpretation of site-group associations (McGeoch, 1998; Niemi & McDonald, 2004; Dufrêne & Legendre, 1997; De Cáceres, Legendre & Moretti, 2010). Evidence from corticolous studies further indicates that bark-associated assemblages can respond to measurable substrate traits, including bark pH, water-holding capacity and, in some systems, host vitality, which strengthens the mechanistic plausibility of substrate-resolved slime moulds indicators in forest monitoring (Everhart, Keller & Ely, 2008; Schnittler, Unterseher & Tesmer, 2006; Schnittler et al., 2016; Vaz et al., 2017; Takahashi & Fukasawa, 2022).

### pH bands: data limitations and cautious interpretation

The pH-resolved view reflects the current availability of measurements. Screening with IndVal E supported mid-band alignment for selected taxa (Table 12). pH results are retained as structured screening evidence that identifies where co-measurement would be most informative and helps to bound plausible pH associations for candidate taxa. pH was not extrapolated or imputed from substrate classes. This treatment is consistent with the wider literature in which pH and water-holding capacity can influence corticolous assemblages but require direct measurement for robust inference (Everhart, Keller & Ely, 2008; Schnittler, Unterseher & Tesmer, 2006; Schnittler et al., 2016; Vaz et al., 2017). Sensitivity checks indicated that modal pH estimates can be resolution-dependent in sparse or boundary cases. These results reinforced that estimated effect magnitudes and predictive scaling were sensitive to resolution and sparsity. Applied use should therefore prioritise rank-based contrasts and cross-validated classification over raw predicted scales.

### Elevational affinities, model adequacy and guidance for application

Effort-adjusted elevational profile summaries provided a complementary stratification axis for interpreting substrate-based signals across heterogeneous forest landscapes. Species-specific modal elevations derived from spline profiles spanned from near sea level to above 1,500 m, with distinct high-elevation modes in *Diderma alpinum* and *Diderma meyerae* and mid-elevation modes in widespread taxa such as *Lycogala epidendrum*, *Arcyria cinerea* and *Metatrichia vesparia* (Fig. 6). Because recording is heterogeneous and presence-only, these modes are interpreted as descriptors that support stratification rather than as precise ecological optima. Elevational structuring of slime mould assemblages has been reported previously and is consistent with covarying climatic and habitat gradients, including in montane systems (Ronikier, Ronikier & Drozdowicz, 2008; Rojas, Valverde & Calvo, 2016). Model checking and sensitivity analyses supported the qualitative robustness of the substrate ranking and reinforced the value of interval- and rank-based interpretation under blocked validation. In applied settings, rank-based contrasts and cross-validated classification should be preferred over raw predicted scales.

## Conclusions

This study demonstrates that slime moulds exhibit consistent, substrate-linked signals that can support bioindication of forest microhabitat (substrate) conditions across Central and Eastern Europe, within an effort-aware, presence-only framework validated in country-blocked partitions. Assemblage differences among substrates were driven chiefly by compositional turnover, whereas standardised record-based richness and evenness remained broadly similar; practical inference should therefore prioritise comparative composition rather than absolute counts.

Substrates were differentiated on a conditional, per-unit-effort scale, with uncertainty captured through rank-based contrasts. Saxicolous material was represented by comparatively few records and is therefore summarised descriptively.

Substrate-linked indicator signals recurred across taxonomic ranks and yielded candidate sets. At the species level, *Cribraria piriformis*, *Amaurochaete atra* and *Arcyria ferruginea* were the strongest candidates for lignicolous conditions, while *Lycogala epidendrum* was frequently recorded on lignicolous substrates. At the genus level, *Reticularia* and *Tubifera* were characteristic of lignicolous material, whereas *Paradiacheopsis* and *Calomyxa* aligned with corticolous conditions; concordant patterns were also evident at family and order levels. pH-associated evidence remains structured screening based on the measured subset (*n* = 151), suggesting mid-band alignment for *Paradiacheopsis solitaria* and prioritising routine co-measurement of pH alongside occurrences.

Effort-adjusted modal elevations provide a complementary stratification axis for indicator deployment, with high-elevation modes observed for *Diderma alpinum* and *Diderma meyerae* and mid-elevation modes typified by *Lycogala epidendrum*, *Arcyria cinerea* and *Metatrichia vesparia*. These descriptors are record-based within the compiled archive and are best used for stratification rather than for inference on precise optima. Elevation can refine substrate-based assessments.

For European bioassessment, validated substrate-based indicators should be prioritised, with elevation incorporated as a stratification covariate. Priorities include systematic recording of pH, improved coverage of under-represented substrates, and continued cross-regional validation of candidate indicators across taxonomic ranks.

## Supplemental Information

10.7717/peerj.21033/supp-1 Supplemental Information 1 Summary of quality-control and harmonisation decision rules relevant to this manuscript.

10.7717/peerj.21033/supp-2 Supplemental Information 2 Cross-validated predictive diagnostics for the no-slopes GLMM (V2; country-blocked folds, K = 3): held-out deviance, RMSE, and calibration slope with 95% confidence limits.

10.7717/peerj.21033/supp-3 Supplemental Information 3 Cross-validated performance by GLMM specification: mean RMSE, Poisson deviance, and calibration slope (country-blocked folds, K = 3).

## Additional Information and Declarations

### Data Availability

The following information was supplied regarding data availability:

The data is available at GBIF:

- Pawłowicz T (2025). Georeferenced Checklist and Occurrence Dataset of Slime Moulds (Eumycetozoa) Across Central and Eastern Europe Emphasising Forest Ecosystems. Biodiversity Data Journal. Occurrence dataset https://doi.org/10.15468/jqukxm accessed *via* GBIF.org on 2026-02-17.

The data is available at Mendeley: Pawłowicz, Tomasz (2026), “Supplementary dataset and replication materials for “Substrate affinities of slime moulds (Eumycetozoa) and their potential as indicators of forest microhabitat conditions””, Mendeley Data, V1, doi: 10.17632/ftbxg2kt9z.1.
