# Supplementary material for: Substrate affinities of slime moulds (Eumycetozoa) and their potential as indicators of forest microhabitat conditions
Source: PeerJ. 2026 May 5;14:e21033. doi: 10.7717/peerj.21033 (PMC13155238; doi:10.7717/peerj.21033)
Supplement: Supplemental Information 1 [file peerj-14-21033-s001.docx]

**Table S1.** Summary of quality-control and harmonisation decision rules relevant to this manuscript.

| Topic | Operational rule in the source archive | Analytical role in this manuscript |
| --- | --- | --- |
| Duplicate screening | Potential duplicates screened at publication/locality granularity using key {authors + year + title + country + verbatimLocality + DOI}; redundant duplicates consolidated whilst distinct sites or dates retained as separate occurrences. | Reduces inflation of record counts arising from repeated reporting of the same occurrence. |
| Taxonomic harmonisation | Accepted scientific names reconciled primarily against Eumycetozoa.com with GBIF Species backbone as fallback; higher taxonomy filled consistently, whilst preserving historical/verbatim usage in the source archive. | Ensures consistent name usage across species, genus, family and order for diversity summaries, mixed models and indicator analyses. |
| Substrate category formation and traceability | substrateCategory assigned using a controlled vocabulary of ten fixed substrate classes with operational definitions and typical examples; microhabitat retained as verbatim free text to preserve original substrate phrasing. | Defines the principal microhabitat axis (Table 1) and enables verification of allocation by inspection of microhabitat against published definitions/examples. |
| Georeferencing deviation limit and coordinate validation | Coordinates supplied only when sites could be localised to within ±10 km; geodeticDatum WGS84; latitude/longitude validated for georeferenced records. | Supports spatial validation and the descriptive record-density map (Fig. 1); coordinates not used as numerical predictors in models. |
| Elevation handling | Elevation parsed and stored as minimumElevationInMeters and maximumElevationInMeters (metres); single values duplicated into both bounds where only one elevation was reported. | Supports elevation profiling and species elevation-response modelling; elevationMid derived here as the mean of minimum/maximum where both present. |
| pH normalisation and rounding | Measured pH normalised from source decimal commas to numeric dots and rounded to two decimals at ingestion; values retained without imputation. | pH used only where measured for descriptive summaries and structured screening; pH discretised here into three bands (≤5.00; 5.01–7.00; ≥7.01). |
