# Supplementary material for: Substrate affinities of slime moulds (Eumycetozoa) and their potential as indicators of forest microhabitat conditions
Source: PeerJ. 2026 May 5;14:e21033. doi: 10.7717/peerj.21033 (PMC13155238; doi:10.7717/peerj.21033)
Supplement: Supplemental Information 2 [file peerj-14-21033-s002.docx]

**Table S2.** Cross-validated predictive diagnostics for the no-slopes GLMM (V2; country-blocked folds, K = 3): held-out deviance, RMSE, and calibration slope with 95% confidence limits.

| Fold | n_holdout | Deviance | RMSE | Calibration slope | LCL | UCL |
| --- | --- | --- | --- | --- | --- | --- |
| 1 | 1836 | 8415.50 | 6.21 | 0.4166 | 0.3850 | 0.4483 |
| 2 | 2098 | 17829.61 | 12.12 | 0.7894 | 0.7686 | 0.8103 |
| 3 | 1683 | 11157.31 | 7.35 | 0.5026 | 0.4719 | 0.5333 |
