# Supplementary material for: Substrate affinities of slime moulds (Eumycetozoa) and their potential as indicators of forest microhabitat conditions
Source: PeerJ. 2026 May 5;14:e21033. doi: 10.7717/peerj.21033 (PMC13155238; doi:10.7717/peerj.21033)
Supplement: Supplemental Information 3 [file peerj-14-21033-s003.docx]

**Table S3.** Cross-validated performance by GLMM specification: mean RMSE, Poisson deviance, and calibration slope (country-blocked folds, K = 3).

| Model | n_folds | mean_RMSE | mean_deviance | mean_calib | calib_LCL | calib_UCL |
| --- | --- | --- | --- | --- | --- | --- |
| V1 baseline: V1: slopes + country | 3 | 7.9513 | 11387.84 | 0.5285 | 0.5004 | 0.5565 |
| V2: no slopes | 3 | 8.5649 | 12467.47 | 0.5696 | 0.5418 | 0.5973 |
